# Validity of the Self-administered Food Frequency Questionnaire Used in the 5-year Follow-Up Survey of the JPHC Study Cohort I: Comparison with Dietary Records for Main Nutrients

**DOI:** 10.2188/jea.13.1sup_51

**Published:** 2007-11-30

**Authors:** Shoichiro Tsugane, Minatsu Kobayash, Satoshi Sasaki

**Affiliations:** Epidemiology and Biostatistics Division, National Cancer Center Research Institute East.

**Keywords:** validity, nutrient, food frequency questionnaire, dietary record

## Abstract

We examined the validity of energy and 16 nutrient intake measurements from a self-administered food frequency questionnaire (FFQ) used in the 5-year follow-up survey of the JPHC study using 28- or 14-day dietary records (DR) as the gold standard. The median (range) correlation coefficients between 16 nutrients measured by FFQ and DR were 0.52 (0.31-0.81) for men and 0.41 (0.22-0.56) for women. The median (range) for energy-adjusted correlation coefficients was 0.40 (0.22-0.82) for men and 0.39 (0.15-0.48) for women. With further adjustment for area, it was 0.41 and 0.35, respectively. The mean percentage of classification into the same categories between the two methods was 33% in men and 30% in women. Only 2% of subjects were classified into the extreme opposite categories. In conclusion, the results suggest that the FFQ can be used in the JPHC Study Cohort I to rank individuals according to the intakes for most of the nutrients examined.

The dietary assessment method is an important issue in prospective epidemiological study on diet and chronic diseases such as cancer and cardiovascular diseases. Considering the long time course of chronic disease development and the mechanistic role of nutrients in diet, the average nutrient intake over one year should be assessed using an appropriate tool. The causative association between nutrient intake and chronic diseases is neither dichotomous (yes or no) nor always linear. The optimal level should be quantitatively explored, so a quantitative assessment of nutrient intake is needed.

Although the long-term dietary record (DR), e.g., 365 days, may be one of most accurate methods for estimating nutrient intake over a given year, it is not appropriate when applied to a large population. A semiquantitative food frequency questionnaire (FFQ), which can estimate the usual level of nutrient intake, was developed and validated in the United States and is now a standard tool in nutritional epidemiology.^1^ We have developed a FFQ for use in the 5-year follow-up survey of the JPHC study, which was based on data obtained from a 3-day DR survey in the same area of the JPHC Study Cohort I.^2^

Here, we examined the validity of energy and 16 nutrient intakes assessed with the FFQ using a 28- or 14-day dietary record (DR).

## MATERIALS AND METHODS

The study design and subject characteristics have been reported elsewhere in this Supplement.^3^ The subjects included in the analysis were 102 men and 113 women who completed 28-day DRs in Iwate, Akita, and Okinawa, and 14-day DRs in Okinawa, and answered the FFQ after the completion of their DRs. The survey method using dietary records and the method for computing nutrient intakes from FFQ have been described elsewhere in this Supplement.^4^^,^^5^ We compared the mean intakes and computed Spearman rank correlation coefficients for energy and 15 nutrients, for which food composition tables are available in the published Standard Tables of Food Composition in Japan, (4^th^ revised edition, by Science and Technology Agency).^6^ We also measured the validity of cholesterol intake, for which a food composition table was developed by substituting the missing values in the published table^7^ using the same method for the developed fatty acid food composition table.^8^ For the computation of intakes from DR, means of 28- or 14-day intakes were used as representative values in this study. Crude and energy-adjusted values were used for computation of the correlation coefficients. A residual model was used for energy-adjustment.^9^

Spearman rank correlation coefficient was used for the correlation analysis because the distribution was skewed in most food groups. In order to validate categorization of the subjects into quintiles by values obtained from the FFQ, we computed the means of intakes obtained from DRs by category as determined according to the nutrient intakes obtained from the FFQ. Moreover, in order to measure the validity of categorization in another way, we computed the number of subjects classified into the same, adjacent, and extreme categories by joint classification by quintiles Because our purpose was to quantify measurement error rather than test a hypothesis, p values were not presented. All the analyses were performed separately on men and women. The computation was performed using the data for the 4 above-mentioned areas combined. We additionally computed the partial correlation coefficients, adjusting for area using dummy variables.

## RESULTS

Table 1 shows the intakes of energy and 16 nutrients assessed with two methods, and their correlation coefficients. For mean intakes, the percent difference varied from -20% for cholesterol to +49% for retinol in men and -11% to +63% for the same food groups in women. The correlation coefficients in crude values varied from 0.31 for total fat to 0.81 for alcohol in men and from 0.22 for total fat to 0.56 for carbohydrate in women. The median was 0.52 in men and 0.41 in women. The correlation coefficients in energy-adjusted values varied from 0.22 for retinol to 0.82 for alcohol in men and from 0.15 for niacin to 0.48 for sodium in women. The median was 0.40 in men and 0.39 in women. The correlation coefficient did not reveal a considerable increase by energy adjustment in most nutrients except for total fat. When further adjustment was made for area, the median partial correlation became 0.41 in men and 0.32 in women for crude and 0.41 in men and 0.35 in women for energy-adjusted intakes.

**Table 1.** Nutrient intakes (g/day) assessed with DR for 28- or 14-days and FFQ in 4 areas and their correlations

Sex	DR			FFQ			%	Spearman correlation			
				
Nutrient	Mean	SD	Median	Mean	SD	Median	difference^1^	Crude	Energy-adjusted^2^	Area-adjusted^3^	Energy- and area-adjusted^2,3^
Men (n=102)											
Energy (kcal/day)	2347	430	1820	2352	732	1862	0	0.55	---	0.36	---
Protein (g/day)	92.9	15.7	76.1	89.5	38.6	71.1	-4	0.50	0.30	0.35	0.34
Total fat (g/day)	59.2	10.6	52.3	66.1	29.6	54.7	12	0.31	0.52	0.30	0.40
Carbohydrate (g/day)	317	81	261	305	101	261	-4	0.71	0.56	0.55	0.58
Alcohol (g/day)	22.6	22.4	0.8	23.8	23.3	0.0	6	0.81	0.82	0.81	0.82
Calcium (mg/day)	623	181	589	685	418	596	10	0.65	0.43	0.50	0.51
Phosphorus (mg/day)	1414	273	1188	1423	595	1183	1	0.61	0 37	0.41	0.46
Iron (mg/day)	12.9	2.6	11.2	12.2	5.6	10.9	-5	0.52	0.49	0.41	0.47
Sodium (mg/day)	5334	1288	4507	5831	2951	4730	9	0.59	0.41	0.34	0.33
Potassium (mg/day)	3218	659	2986	3309	1596	2802	3	0.52	0.39	0.41	0.48
Retinal (mg/day)	439	471	206	653	602	427	49	0.40	0.22	0.34	0.19
Carotene (mg/day)	3274	1305	2885	3814	3126	3320	16	0.38	0.36	0.38	0.29
Vitamin B1 (mg/day)	1.32	0.29	1.11	1.27	0.54	1.05	-4	0.49	0.40	0.43	0.41
Vitamin B2 (mg/day)	1.55	0.36	1.39	1.78	0.81	1.52	15	0.54	0.34	0.44	0.43
Niacin (mg/day)	21.9	4.0	16.5	21.0	8.8	15.8	-4	0.42	0.35	0.37	0.35
Vitamin C (mg/day)	129	39	127	166	118	157	29	0.44	0.42	0.44	0.39
Cholesterol (mg/day)	418	97	404	334	155	320	-20	0.42	0.33	0.36	0.38
Median								0.52	0.40	0.41	0.41

Women (n=113)											
Energy (kcal/day)	1820	316	891	2018	862	751	11	0.44	---	0.32	---
Protein (g/day)	76.2	13.1	30.9	82.7	47.1	34.5	9	0.41	0.27	0.32	0.29
Total fat (g/day)	52.9	9.8	24.3	64.5	37.5	24.5	22	0.22	0.46	0.21	0.32
Carbohydrate (g/day)	257	58	135	275	98	84	7	0.56	0.37	0.42	0.33
Alcohol (g/day)	1.62	2.90	0.00	1.52	7.15	0.00	-6	0.51	0.42	0.47	0.47
Calcium (mg/day)	600	166	213	699	418	191	17	0.53	0.47	0.41	0.45
Phosphorus (mg/day)	1172	222	454	1321	667	502	13	0.49	0.42	0.36	0.41
Iron (mg/day)	11.3	2.5	5.1	12.1	7.3	4.2	7	0.41	0.33	0.30	0.32
Sodium (mg/day)	4652	1143	2135	5437	3308	1269	17	0.55	0.48	0.31	0.35
Potassium (mg/day)	2949	657	1454	3344	1922	1249	13	0.40	0.31	0.32	0.35
Retinol (mg/day)	370	425	47	603	699	61	63	0.35	0.43	0.32	0.41
Carotene (mg/day)	3184	1262	2870	4105	3029	3358	29	0.31	0.33	0.29	0.25
Vitamin B1 (mg/day)	1.12	0.24	0.54	1.24	0.65	0.44	10	0.31	0.41	0.27	0.44
Vitamin B2 (mg/day)	1.38	0.31	0.50	1.72	0.88	0.57	25	0.43	0.45	0.34	0.46
Niacin (mg/day)	16.9	3.2	6.8	18.3	11.3	7.2	8	0.27	0.15	0.25	0.18
Vitamin C (mg/day)	137	50	127	192	159	156	40	0.31	0.22	0.28	0.16
Cholesterol (mg/day)	356	87	354	316	168	306	-11	0.31	0.35	0.28	0.34
Median								0.41	0.39	0.32	0.35

^1^(FFQ mean - DR mean)/DR mean (%).

^2^Energy intake was adjusted for residual model.

^3^Area was adjusted for dummy variables.

For n=102, r>0.20 = p<0.05, r>0.26 = p<0.01, r>0.33 = p<0.001. For n=113, r>0.19 = p<0.05, r>0.25 = p<0.01, r>0.31 = p<0.001.

Table 2 shows mean nutrient intakes assessed with DR within a quintile of intake assessed with FFQ. The mean intake in the highest quintile was 1.5 times or more higher than in the lowest quintile for retinol (3.10), calcium (1.76), carbohydrate (1.66), carotene (1.59), vitamin C (1.54), sodium (1.51) and vitamin B2 (1.51) in men, and in the lowest quintile for retinol (1.64), calcium (1.54) and carotene (1.51) in women. A steady increase in mean intake of 16 nutrients from the lowest to the highest quintile was observed in energy, carbohydrate, calcium, phosphorus, iron, potassium, vitamin B_2_, niacin, and cholesterol in men, and in energy, carbohydrate, calcium, phosphorus, carotene, and vitamin B_2_ in women.

**Table 2.** Mean intake of total energy and 15 nutrients from DR within quintile of intake determined by FFQ

Sex	Quintile of nutrient intake according to FFQ													
	
Nutrient	Lowest		Second			Third			Fourth			Highest		
				
	Mean	SD	Mean	SD		Mean	SD		Mean	SD		Mean	SD	
Men (n=102)	(n=20)		(n=21)			(n=20)			(n=21)			(n=20)		
Energy (kcal/day)	2064	401	2145	381		2353	332		2459	337	**	2716	391	***
Protein (g/day)	79.6	14.4	88.5	13.3		98.6	13.5	***	93.4	12.5	**	104.7	13.3	***
Total fat (g/day)	54.0	13.0	58.3	8.5		58.2	9.6		60.2	9.2		65.4	10.0	**
Carbohydrate (g/day)	241.2	52.5	286.3	68.4		301.6	31.0	**	355.5	68.1	***	399.4	72.8	***
Calcium (mg/day)	448.6	114.9	555.3	165.0		622.1	94.3	***	700.4	151.3	***	789.3	164.7	***
Phosphorus (mg/day)	1126	182	1348	263	**	1460	184	***	1499	207	***	1639	236	***
Iron (mg/day)	11.3	2.1	11.9	2.4		12.6	2.9		13.8	1.7	**	14.8	2.5	***
Sodium (mg/day)	4263	917	4861	1173		5621	1162	**	5484	836	***	6454	1251	***
Potassium (mg/day)	2760	541	3084	717		3093	439		3337	416	**	3814	671	***
Retinol (mg/day)	185.7	180.9	371.0	297.7		583.0	548.5	*	482.2	349.8		574.8	713.6	*
Carotene (mg/day)	2544	951	3071	1185		3529	1594	*	3231	1044		4035	1299	***
Vitamin B1 (mg/day)	1.13	0.29	1.28	0.17		1.26	0.19		1.34	0.19	*	1.59	0.35	***
Vitamin B2 (mg/day)	1.20	0.23	1.48	0.33	*	1.62	0.31	***	1.67	0.30	***	1.81	0.34	***
Niacin (mg/day)	18.9	3.3	21.4	4.6		22.1	3.0	*	22.6	2.9	**	24.3	4.0	***
Vitamin C (mg/day)	103.9	32.0	140.9	47.7		117.9	25.5	*	138.4	35.9	**	159.6	40.5	***
Cholesterol (mg/day)	360.4	100.1	382.1	76.0	*	430.5	71.7	*	431.9	104.6	*	484.6	86.5	***

Women (n=113)	(n=22)		(n=23)			(n=23)			(n=23)			(n=22)		
Energy (kcal/day)	1611	217	1709	329		1832	221	*	1917	323	**	2029	311	***
Protein (g/day)	68.0	10.2	74.7	15.3		74.1	9.9		80.2	12.8	**	83.9	11.6	***
Total fat (g/day)	49.7	10.0	52.6	10.4		52.3	10.4		55.1	10.0		54.9	7.9	
Carbohydrate (g/day)	213.2	52.1	234.8	39.9		256.3	45.6	**	279.4	41.4	***	300.9	67.5	***
Calcium (mg/day)	481.4	131.5	543.0	154.8		574.6	111.3		658.1	144.1	***	742.4	161.1	***
Phosphorus (mg/day)	1000	163	1124	240		1174	177	*	1238	209	***	1323	189	***
Iron (mg/day)	9.5	2.1	10.7	2.1		11.7	2.5	**	11.6	1.9	**	12.8	2.5	***
Sodium (mg/day)	3512	745	4315	933	*	4954	1135	***	5315	937	***	5136	959	***
Potassium (mg/day)	2577	509	2836	737		2830	437		3227	694	**	3273	634	**
Retinal (mg/day)	335.9	364.4	281.3	474.7		284.5	205.1		402.3	375.8		550.3	592.0	
Carotene (mg/day)	2612	1022	3034	1084		3085	1143		3284	1383		3933	1366	**
Vitamin B1 (mg/day)	0.99	0.18	1.15	0.22		1.08	0.18		1.19	0.26		1.21	0.30	*
Vitamin B2 (mg/day)	1.19	0.31	1.32	0.28		1.33	0.25		1.47	0.28	**	1.59	0.28	***
Niacin (mg/day)	15.5	2.7	16.3	3.6		17.1	2.6		18.1	3.4		17.7	3.3	*
Vitamin C (mg/day)	120.7	46.1	132.3	62.9		139.4	47.1		154.7	42.1		150.2	49.1	
Cholesterol (mg/day)	308.4	95.0	339.3	68.6		370.0	88.1	*	390.7	78.5	**	371.5	84.9	*

Significance of Dunnett test of ANOVA with the lowest quintile as reference: * p<0.05, ** p<0.01, *** p<0.001.

Table 3 shows the comparison of FFQ with DR based on joint classification by quintile. Each classification of the categories was presented in the Appendix to this Supplement. The mean percentage of classification into the same categories between the two methods was 33% in men and 30% in women. Only 2% of subjects were classified into the opposite extreme categories.

**Table 3.** Comparison of FFQ with DR for nutrients based on joint classification by quintile (%)

	Men (n=102)			Women (n=113)			
	
	Same	Adjacent	Extreme	Same	Adjacent	Extreme
	category	category	category	category	category	category
Energy (kcal/day)	34	75	2	32	70	1
Protein (g/day)	37	73	2	21	68	2
Total fat (g/day)	26	65	4	31	62	5
Carbohydrate (g/day)	43	86	0	37	69	3
Calcium (mg/day)	41	80	0	36	73	1
Phosphorus (mg/day)	39	76	1	34	70	1
Iron (mg/day)	31	72	1	26	65	0
Sodium (mg/day)	36	72	0	35	73	2
Potassium (mg/day)	32	72	2	32	68	2
Retinol (mg/day)	23	68	3	23	64	4
Carotene (mg/day)	31	72	3	27	65	2
Vitamin B1 (mg/day)	37	69	3	30	60	2
Vitamin B2 (mg/day)	33	70	0	34	67	2
Niacin (mg/day)	29	72	2	24	64	5
Vitamin C (mg/day)	30	70	2	29	63	4
Cholesterol (mg/day)	27	74	4	28	62	6

Each classification of categories is presented in the Appendix to this Supplement

## DISCUSSION

We examined the validity of FFQ using 28- or 14-day DR data. The median correlation coefficients observed in this study were similar to or slightly lower than those in previously developed and validated dietary assessment questìonnaires in Japan.^10^^-^^12^

The validity in crude intakes was better than for energy-ajusted intakes both in men and women (Table 1), something hardly ever observed in the previous validation studies.^13^^-^^15^ However, when adjustment was made for area, the difference in crude and energy-adjusted intakes almost disappeared. In contrast to the present study, most of the previous validation studies have been conducted in one area.^13^^-^^15^

The validity was better in men than in women for most of the nutrients examined. This was unexpected because women in Japanese populations do most of the food preparation and cooking. However, the greater validity in men than in women has already been observed in Japanese populations, ^13^^,^^15^ not only in Western populations.^16^ This type of structured questionnaire, which simplifies daily dietary habits as much as possible, may be easier for male subjects to answer because they are not so interested in dietary habits. Female subjects, on the other hand, are more keen about their diets.

Ethanol, carbohydrate, and calcium, and probably phosphorus, potassium, and vitamin B2 as well, were nutrients whose values were reliably assessed with this FFQ. Although retinol showed a low validity in men, this result seemed inconclusive because of the wide within-individual variation.^17^ The reason for the low validity of niacin in women is unclear. The low validity for niacin was also observed in one previous dietary assessment questionnaire in Japanese women.^14^ The low validity of vitamin C in women may be due to the wide seasonal variation of this nutrient, but it remains to be clearly explained.^18^

Although the mean intakes were similar between the intake assessed with FFQ and DR for energy, protein, carbohydrate, and some other nutrients in men, the much wider standard deviation suggested that the absolute intake estimated by this FFQ at the individual level needs to be used with caution. The mean intakes were generally overestimated in the FFQ in women. This may be partly due to the standard portions/units of foods (except rice and miso-soup) used for calculation, which did not consider possible sex-differences.

In conclusion, we observed moderate ability to rank individuals for the nutrients examined when intakes were assessed with DR as the gold standard. However, the validity varied between nutrients examined, and was generally better in men than in women. The results on disease-nutrient intake associations reported in subsequent communications should be cautiously interpreted in light of the results of the present study.

## Appendix.

Contingency table for joint classification of nutrient intake assessed by FFQ and DR
